# Inflammatory and Ischemic Post Liver Transplant Complications Mimic Malignancy on ^18^F-FDG PET/CT

**DOI:** 10.4274/mirt.03371

**Published:** 2018-02-01

**Authors:** William Makis, Anthony Ciarallo, Stephan Probst

**Affiliations:** 1 Cross Cancer Institute, Department of Diagnostic Imaging, Edmonton, Canada; 2 McGill University Health Centre, Department of Nuclear Medicine, Montreal, Canada; 3 Jewish General Hospital, Department of Nuclear Medicine, Montreal, Canada

**Keywords:** Liver transplant, complications, pitfall, artifact, ^18^F-fluorodeoxyglucose, positron emission tomography

## Abstract

A 65-year-old male patient with a one year history of liver transplantation was referred for an ^18^F-fluoro-2-deoxy-D-glucose (^18^F-FDG) positron emission tomography/computed tomography (PET/CT) to rule out post transplant lymphoproliferative disease. Multiple foci of intense abnormal ^18^F-FDG uptake were seen in the transplanted liver which were concerning for malignancy. Explantation of the liver approximately 1 month following the PET/CT revealed multiple inflammatory and ischemic changes including large bile duct necrosis, acute cholangitis, bile duct obstruction changes and periportal fibrosis, with no evidence of malignancy. We present the ^18^F-FDG PET/CT image findings of this case.

## Abstract (tr)

Altmış beş yaşında bir erkek hasta karaciğer transplantasyonundan bir yıl sonra post transplant lenfoproliferatif hastalık açısından değerlendirilmek üzere ^18^F-fluoro-2-deoxy-D-glucose (^18^F-FDG) pozitron emisyon tomografisi/bilgisayarlı tomografi (PET/BT) için yönlendirildi. Transplante karaciğerde malignite şüphesi uyandıran multipl yoğun ^18^F-FDG tutulumu saptandı. PET/BT’den 1 ay sonra yapılan karaciğer eksplantasyonunda multipl enflamatuvar ve iskemik değişiklikler, safra kanalı nekrozu, akut kolanjit, safra yolu obstrüksiyonuna bağlı değişiklikler ve periportal fibrozis saptandı, malignite bulgusu görülmedi. Bu olgunun ^18^F-FDG PET/BT görüntüleme bulgularını sunuyoruz.

## Figures and Tables

**Figure 1:**
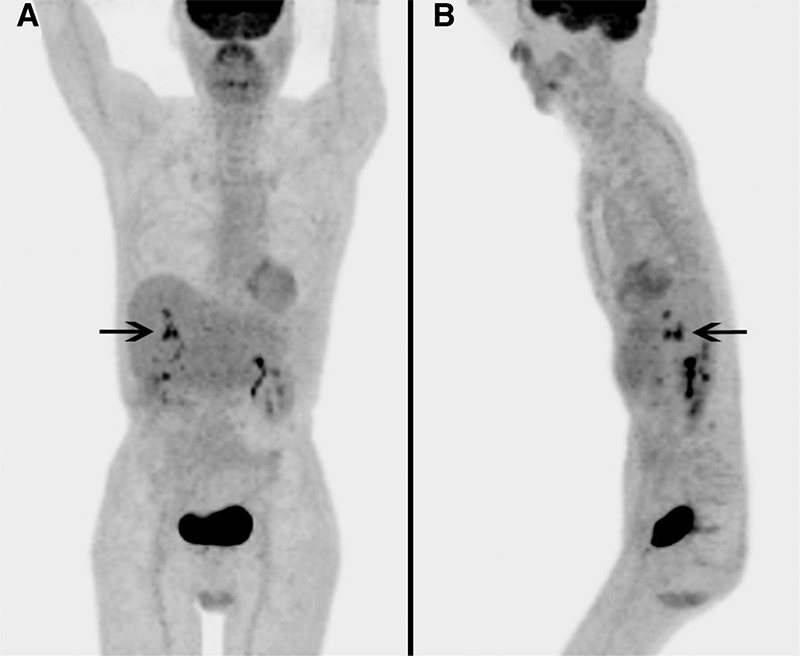
A 65-year-old male had a liver transplant one year prior, for hepatitis B cirrhosis and hepatocellular carcinoma (HCC) with a 3.5 cm lesion in the left lobe and a 4 cm lesion in the right. For several months prior to positron emission tomography/computed tomography (PET/CT), the patient had persistently elevated liver function tests but multiple transjugular biopsies showed no evidence of transplant rejection. Seven endoscopic retrograde cholangiopancreatographies (ERCPs) were performed as the patient developed recurring stenoses and strictures of intrahepatic bile ducts, with failed attempts of cannulation, balloon dilation and stent replacements. The patient tested positive for Epstein-Barr virus and underwent PET/CT to rule out post transplant lymphoproliferative disease (PTLD). Maximum intensity projection images showed multiple foci of intense ^18^F-FDG uptake in the liver in segments 5, 6, 7 and 8, with maximum standardized uptake value of 9.0, concerning for malignancy.

**Figure 2:**
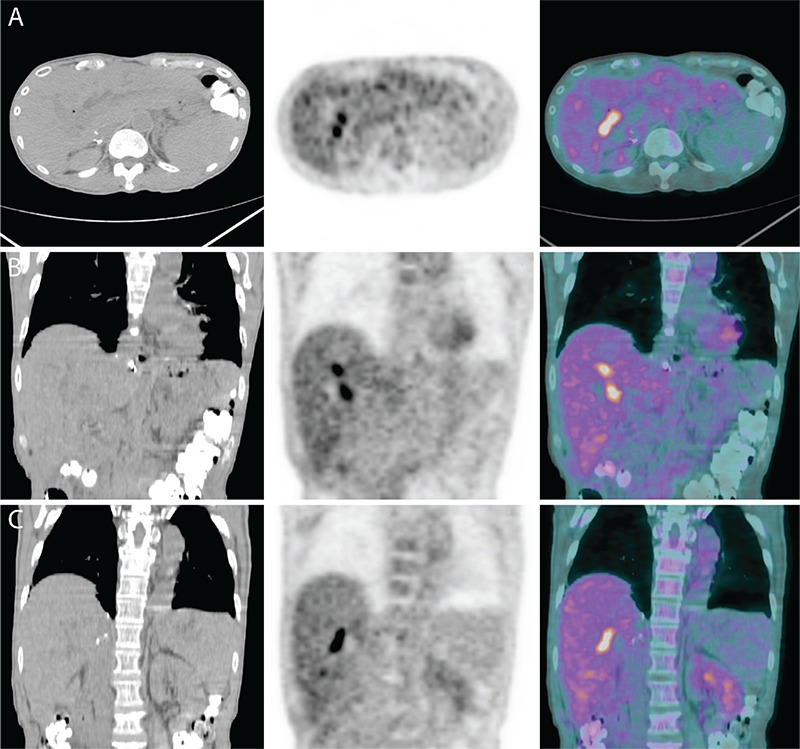
Transaxial and coronal views of the PET/CT fusion images showed multiple foci of intense ^18^F-FDG uptake in the liver, although no obvious mass lesions were seen on the CT. An ERCP done 1 month after PET/CT showed multiple strictures involving the common hepatic duct and its intrahepatic segments associated with stones, debris and a failed right hepatic biliary stent. The liver was explanted and histopathological evaluation showed a cirrhotic liver weighing 1260 g measuring 20x14.5x7.2 cm. The surface of the liver revealed homogeneous macronodular appearance with absence of infarctions. Ducts of the right and left lobe appeared dilated, and sectioning showed large bile duct necrosis, acute cholangitis, bile duct obstruction changes and periportal fibrosis (stage 2/4). There was no evidence of malignancy within the explanted liver. Liver transplantation is a standard treatment for patients with end stage liver disease. Biliary complications of liver transplantation remain a significant problem with bile leak and stricture rates of ~20%, majority of them being related to biliary anastomosis (1,2). Re-transplantation is required following the development of ischemic type biliary strictures or ischemic cholangiopathy which is defined as intra or extra-hepatic biliary stricture in the presence of a patent hepatic artery (3). The only ^18^F-FDG positive liver transplant complications reported in the literature are PTLD and recurrent HCC (4,5). ^18^F-FDG PET/CT is widely used to assess for extrahepatic metastases prior to liver transplantation (6). PET/CT has also been found to be useful as a predictive parameter for evaluation of early HCC recurrence after liver transplantation (7). In light of the increasing use of PET/CT in liver transplant patients, it is important to be aware of lesions that mimick malignancy. In our case, foci of increased ^18^F-FDG uptake in the transplanted liver were concerning for malignancy but were found to be benign inflammatory and ischemic changes including large bile duct necrosis, acute cholangitis, bile duct obstruction changes and periportal fibrosis. In non-transplanted liver, several non-malignant processes have been described to take up ^18^F-FDG including: intrahepatic cholestasis (8), acute cholangitis (9,10), sclerosing cholangitis (11), liver abscess (12,13), hepatic pseudotumor (14), and hepatic sarcoidosis (15).
